# Formate Dehydrogenase Mimics as Catalysts for Carbon Dioxide Reduction

**DOI:** 10.3390/molecules27185989

**Published:** 2022-09-14

**Authors:** Thibault Fogeron, Yun Li, Marc Fontecave

**Affiliations:** Laboratoire de Chimie des Processus Biologiques, UMR 8229 CNRS, Collège de France, Paris Sorbonne University, 11 Place Marcelin Berthelot, CEDEX 05, 75231 Paris, France

**Keywords:** formate dehydrogenases, structural models, functional models, dithiolene complexes, carbon dioxide reduction

## Abstract

Formate dehydrogenases (FDH) reversibly catalyze the interconversion of CO_2_ to formate. They belong to the family of molybdenum and tungsten-dependent oxidoreductases. For several decades, scientists have been synthesizing structural and functional model complexes inspired by these enzymes. These studies not only allow for finding certain efficient catalysts but also in some cases to better understand the functioning of the enzymes. However, FDH models for catalytic CO_2_ reduction are less studied compared to the oxygen atom transfer (OAT) reaction. Herein, we present recent results of structural and functional models of FDH.

## 1. Introduction

The storage of diluted and intermittent sources of energy such as solar and wind energy is a great challenge in the 21st century. It can be accomplished by the conversion of solar or wind electricity into chemical energy, via the formation of chemical bonds that can durably store the energy. For this reason, the reduction of CO_2_ is particularly appealing since, through its transformation into a variety of energy-dense carbon compounds, it not only allows energy storage but also gives access to fuels, allowing the use of renewable energy as a source of energy without an important shift in technology. The reduction of CO_2_ can also be used as a carbon feedstock to form precursors for organic synthesis, leading to valuable organic compounds useful for the chemical industry [1,2].

**Figure 1:**
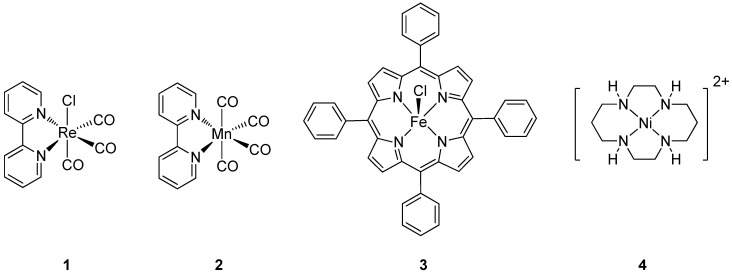
Some of the most-known molecular catalysts for CO_2_ reduction.

CO_2_ is a thermodynamically stable molecule. Furthermore, its reduction implies multi-electron and multi-proton transfers which results in very low kinetics. As a consequence, catalysts are absolutely needed. If heterogeneous materials are generally considered more stable and more efficient catalysts for CO_2_ reduction, molecular metal complexes can offer more tunability and selectivity [3]. For these reasons, the molecular approach has been extensively developed over the last decades. Among the most remarkable and studied complexes reported in the literature, one can mention Re- and Mn-bipyridine complexes (**1** and **2**), metal complexes using N-based macrocycles, such as porphyrins **3**, phtalocyanins and quaterpyridines, and Ni-cyclam complex **4** (Figure 1) [4]. Nevertheless, the library of complexes able to catalyze the reduction of CO_2_ efficiently and selectively remains narrow, especially if we compare it with molecular complexes for hydrogen production.

In order to expand this library and develop catalysts with non-noble metals, the bioinspired approach, yet in its infancy, is likely to be useful. In fact, while a number of enzymes have shown the ability to catalyze the conversion of CO_2_ into CO or formic acid using unique metal active sites, very little has been done to synthesize bioinspired complexes mimicking these active sites and evaluate their catalytic activity. In this review, we focus on the models based on molybdenum- and tungsten-dependent formate dehydrogenases [5]. We show that this bioinspired approach can lead to new classes of interesting molecular catalysts for CO_2_ photo- and electro-reduction.

## 2. Formate Dehydrogenases (FDHs)

FDHs catalyze the reversible transformation of CO_2_ to HCOOH according to a two-electron redox process (Equation (1)). There are two classes of FDHs: NAD-dependent ones that do not contain any metal ion, and metal-dependent ones that possess a mononuclear molybdenum (or tungsten) center. Several excellent reviews have described the different aspects of this enzyme in detail [5,6,7,8]. We will herein focus on the metal-dependent FDH and settle for a short summary.


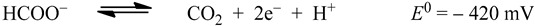
 (1)

**Figure 2:**
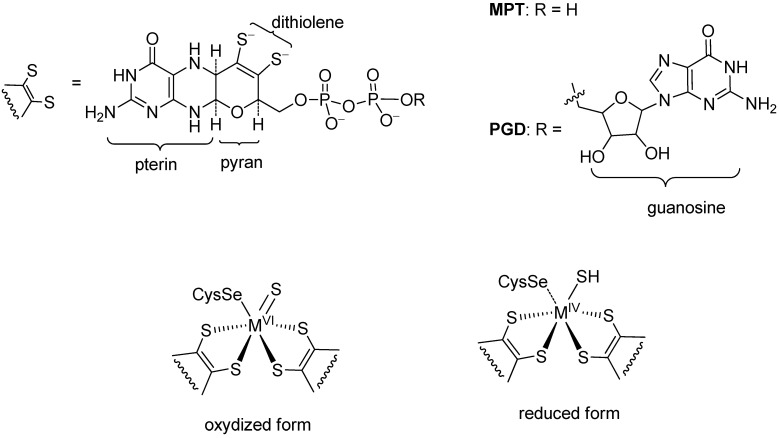
Structures of PGD and MPT (**top**) and active site of FDHs: M = Mo/W (**bottom**). The presence of the selenocysteine ligand in the reduced form is debated.

The structures of several FDHs from different organisms have been elucidated by X-ray diffraction [9,10,11,12]. The Mo (or W) atom is coordinated by two guanidine phosphate-esterified pyranopterin cofactors called PGT (Figure 2, top). The [Mo(PGT)_2_] (or [W(PGT)_2_]) structure is well conserved in FDHs from different organisms and during the catalytic cycle. The cofactor without the guanosine moiety is called molybdopterin (MPT, Figure 2, top). MPT is a highly unstable organic molecule and has never been isolated without the protein backbone. It is composed of a pterin with a fused pyran ring carrying a dithiolene chelate (Figure 2, top). The redox state of the Mo (or W) atom varies from +VI to +IV during the catalytic cycle. Additional apical ligands are present; however, their exact coordination configuration remains controversial. It is generally admitted that, in the oxidized form, the Mo^VI^/W^VI^ atom is coordinated by a selenocysteine (in a few cases a cysteine) residue and a terminal sulfido ligand (Mo/W=S) that is essential for catalytic activity. However, at first and for a long time, this ligand was thought to be a terminal oxo ligand. It was only recently (2006) that this result was reinterpreted by Romão et al., who determined it to be a sulfido ligand [12]. The reduced form of the enzyme is still a subject of controversy: the Mo^IV^/W^IV^ center is presumably coordinated with a terminal hydrosulfido ligand (Mo/W˗SH), along with the selenocysteine (or cysteine) residue (Figure 2, bottom). However, some publications suggest that the selenocysteine (or cysteine) residue is no longer present and the metal center is pentacoordinated with an SH ligand [12].

**Scheme 1:**
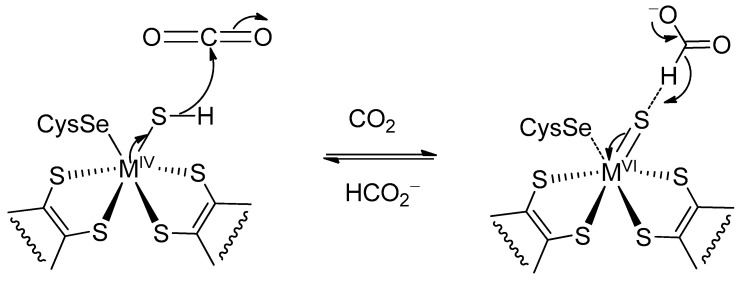
One of the possible proposed mechanisms of FDHs (M = Mo/W).

Recent studies show that there is no clear-cut experimental evidence that formate/carbon dioxide coordinates to the metal center during the catalytic oxidation/reduction process. Furthermore, the formation of an M(IV)^_^H hydride species could not be detected during the process of the reduction of carbon dioxide. Due to the fact that there are some ambiguities over the reduced form, the mechanism of this enzyme is not unanimously established. We herein show one mechanism among other proposals with M(IV)-SH for hydride transfer during the carbon dioxide reduction step (Scheme 1) [13]. More experimental studies are needed to unambiguously determine the mechanism.

## 3. Structural Models of FDH

As mentioned earlier, the exact nature of the axial ligand and its role in the mechanism has been only recently emphasized [7]. It was thought that FDHs catalyze oxygen transfer reactions, as most molybdenum and tungsten enzymes do, which was compatible with the first crystallographic structure of an enzyme of this family having M^VI^=O/M^IV^˗OH as the axial ligands. This is probably why, so far, most models were synthesized with a terminal oxo ligand.

As a result, very few M^VI^=S/M^IV^˗SH complexes have been developed (M = Mo/W). 

The main challenges for the development of synthetic models of the active site of FDHs resided in the stabilization of bis-dithiolene Mo/W-complexes. The first models of MPT-containing enzymes were synthesized as models of oxotransferases such as DMSO reductase (DMSOR). Most of this work was done by the group of Richard Holm in the 90s [14], who developed various synthetic routes to obtain bis-dithiolene Mo(W)-oxo complexes. Quite simple dithiolene ligands were used. Other groups also contributed to this effort with simple dithiolene ligands [15,16,17,18]. 

Two other challenges to be addressed for close mimics of FDHs regarded the nature of the axial ligands and the structure of the dithiolene ligands. 

### 3.1. Variation of the Axial Ligands

**Scheme 2:**
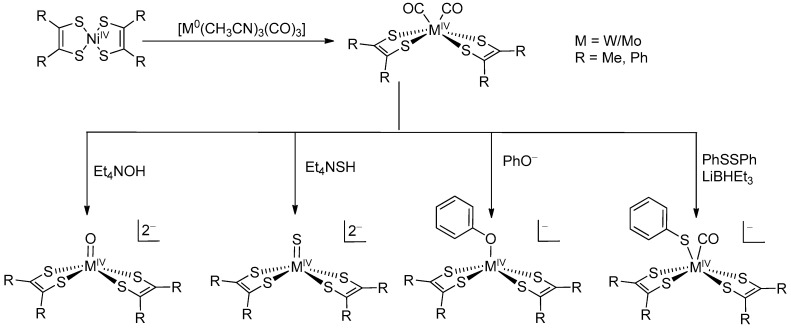
Synthetic route of bis-dithiolene Mo(W) complexes with different axial ligands developed by Holm and co-workers.

With simple dithiolene ligands (R = Me, Ph), Holm et al. developed a method allowing the introduction of different axial ligands thanks to the substitution of carbon monoxide ligands on molybdenum or tungsten carbonyl complexes [19,20,21,22,23]. The precursors Mo/W(dithiolene)_2_(CO)_2_ were prepared by a trans-metalation reaction from a Ni(IV)-bisdithiolene complex. Unfortunately, the overall yields for the preparation of such complexes are quite low, which is a strong limitation if one wants to use more elaborate ligands. It seems that this methodology can only be applied to simple dithiolene ligands with Me and Ph groups (Scheme 2). More recently, Elvers et al. synthesized similar complexes thanks to the photochemical deprotection of 1,3-Dithiol-2-ones and in situ complexation [24]. In this case, the yields remained modest (max 20 %) and aliphatic dithiolenes ligands could also be used. 

### 3.2. Pyrazine Containing Ligands

**Figure 3:**
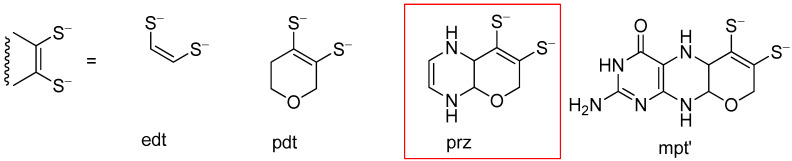
Prz as the minimum required ligand according to the DFT calculations.

However, the active site of FDH cannot be modeled only by its first coordination sphere, as the pterin part of the ligands plays an important role as a proton relay and also as a mediator of the red-ox properties of the active site. Schulzke and co-workers performed DFT calculations with model complexes Mo/W(O)(dithiolene)_2_ and showed that in order to accurately reproduce the molecular/electronic properties of MPT, a simple dithiolene ligand was not appropriate even within a pyran ring and that the minimal ligand should be a fused pyran-tetrahydropyrazine-functionalized dithiolene, denoted prz. The third pyrimidine cycle seems not to be mandatory (Figure 3) [25]. 

Hence, in the following paragraph, we will describe only ligands bearing such a structure, with different oxidation states for the pyrazine cycle. The formation of a pyran cycle fused with a pyrazine unit represents the most challenging synthetic step. 

**Scheme 3:**
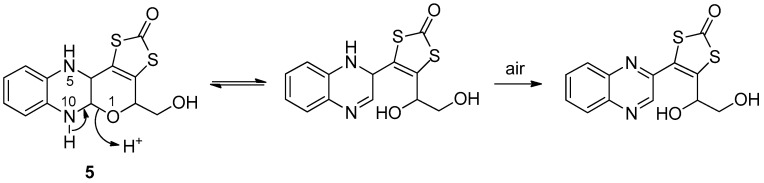
Pyran ring scission according to Joule and co-workers.

One of the earliest and most elaborate syntheses of MPT-like ligands was developed by the groups of Joule and Garner at the end of the 90s [26,27,28,29,30]. It was specified that without the protection of the secondary amine N10, **5** was not stable and the pyran ring could open up in a proton-assisted reaction (Scheme 3) [27]. In contrast, MPT is stable in the tricyclic form within FDHs, possibly because the active site does not contain a proton exchange site that favors pyran ring opening. One should note that, under very specific conditions, during protein crystallization, for example, open forms of MPT have been obtained, reflecting the sensitivity of such pterin-pyran-dithiolene molecules [10,31,32]. However, the physiological relevance of these open forms is not established [33].

**Scheme 4:**
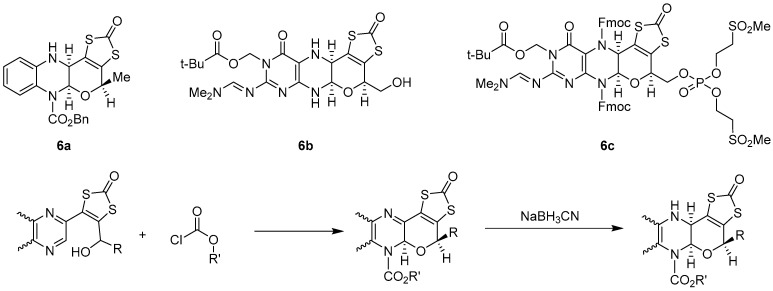
MPT-like ligands synthesized by Joule and co-workers.

In all the syntheses, the formation of the pyran ring was achieved by using a chloroformiate reagent to activate the nitrogen on the pyrazine cycle, after the protection of the dithiolene moiety. This activation also allowed the protection of the amine N10 and thus avoided an opening of the pyran cycle. The second imine of the cycle was then reduced and the resulting amine could be protected to prevent reoxidation. In these remarkable syntheses, even the stereochemistry of the asymmetric carbons was matching with the natural ligand. Subsequently, **6a**, **6b,** and **6c** were obtained (Scheme 4). Unfortunately, no molybdenum or tungsten complexes were described with these ligands. Nevertheless, CoCp(dithiolene) (Cp = cyclopentadienyle) complexes were obtained, proving the possibility for the molecules to coordinate a metal center once deprotected.

**Scheme 5:**
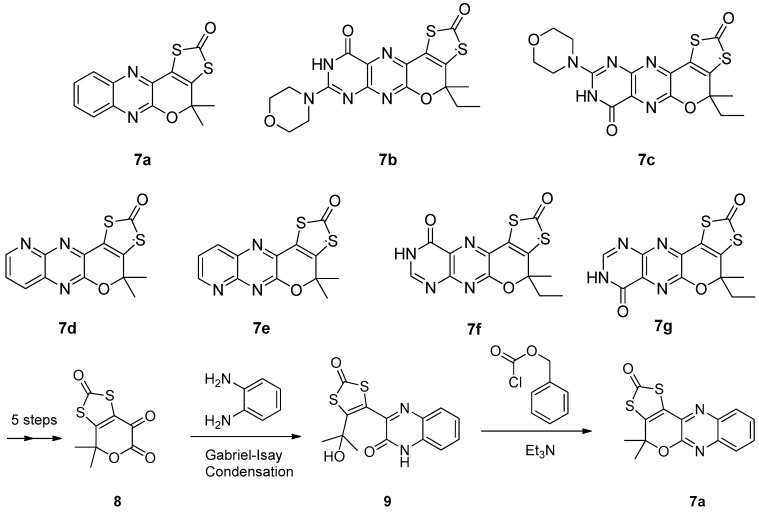
MPT-like ligands synthesized by Basu and co-workers.

Later, Basu’s group obtained a family of dithiolene ligands containing the prz moiety **7a**–**7g** (Scheme 5). Here again, after the protection of the dithiolene function in **8**, a chloroformate activation through an intramolecular reaction between an amide and an alcohol in **9** allowed the formation of the pyran cycle **7**. However, in these cases, the carbamate derivatives were not obtained, and a fully oxidized pyrazine cycle was instead formed (Scheme 5). The main drawback of this strategy was the relatively low global yield, especially when the di-amine used in the Gabriel-Isay condensation was asymmetric due to the poor regioselectivity of this step. Again, no tungsten or molybdenum complexes using these ligands were reported [34,35].

**Scheme 6:**
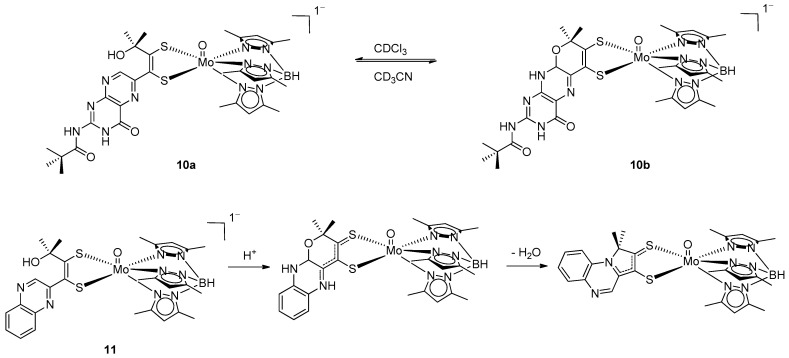
Solvent-dependent equilibrium between the open chain forms and the cyclized pyran forms.

Burgmayer et al. used a rather original approach to prepare a closed pyran cycle. They observed tautomerism between an open and a closed pyran cycle **10a** and **10b** directly on a molybdenum complex with a dithiolene ligand and a tris(3,5-dimethylpyrazolyl)hydroborate (Tp*) chelate (Scheme 6) [36,37]. The spontaneous cyclization depends upon the polarity of the solvent in which the complex was studied. This reaction was also studied with a simpler ligand **11** (Scheme 6) [38]. In this case, the cyclization was not spontaneous but was promoted by the addition of trifluoroacetic acid (TFA). Unfortunately, the closed form was not stable, and the dehydration of the ligand was observed, yielding to a pyrrolo-quinoxaline ligand.

This study brings interesting insights into the catalytic site of FDH. However, the nature of the Tp* ligand is quite different from the natural ligand. Moreover, due to the equilibrium phenomenon, a mixture of two forms could be obtained, so it is hard to envisage organic reactions on the ligand to stabilize the pyran form once it is formed. Thus, these systems are not appropriate for catalytic studies. 

**Scheme 7:**
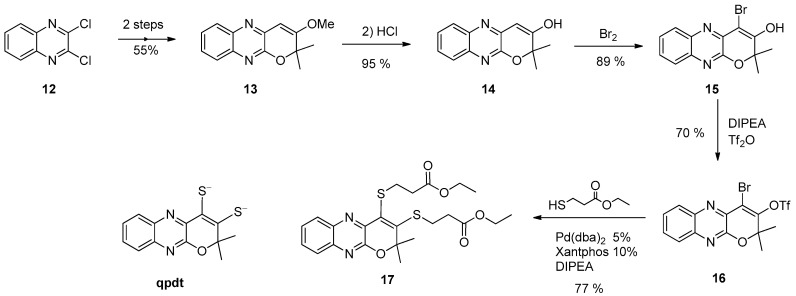
Synthesis of the protected ligand **17** and structure of ligand qpdt.

In a quest for the bioinspired catalyst for CO_2_ reduction, we developed a prz-containing ligand with an oxidized pyrazine cycle similar to the one obtained by Basu et al. (Scheme 5). However, we adopted a new synthetic strategy, wherein we chose to first prepare the tricyclic skeleton and then introduce a protected dithiolene moiety [39,40]. Starting from 2,3-dichloroquioxaline **12**, the tricyclic molecule **13** was prepared in two steps according to a reported method [41]. The enol ether was hydrolyzed to the corresponding enol **14**, which was functionalized by bromination to **15** and activated by triflation to give **16**. Finally, a double cross-coupling reaction catalyzed by palladium allowed the introduction of the protected dithiolene moiety. The protected ligand **17** was prepared in a straightforward and easily scalable way (Scheme 7). 

**Scheme 8:**
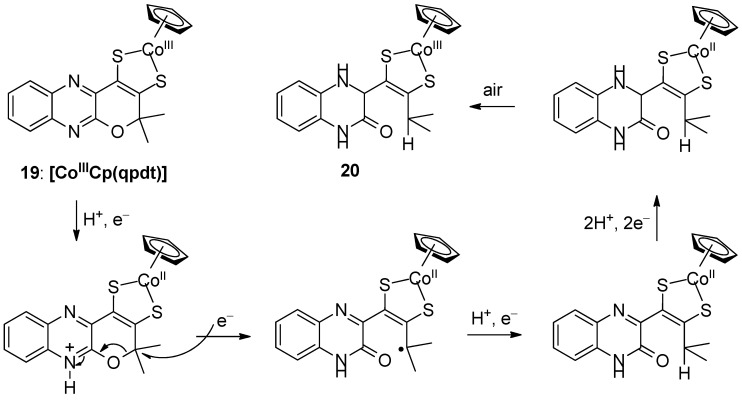
Electro-reduction of [Co^III^Cp(qpdt)] resulting in an unusual ring scission.

**Figure 4:**
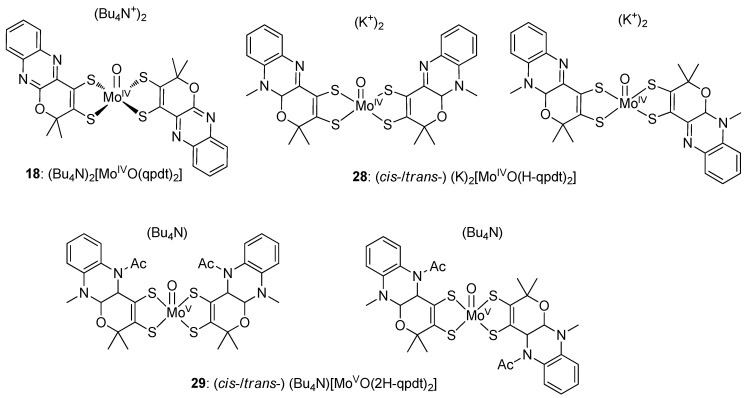
Structures of complexes **18**, **28**, and **29**.

After the deprotection of **17**, the quinoxaline-pyran-fused dithiolene ligand qpdt (Scheme 7) could be obtained and directly used for complexation. The biomimetic complex (Bu_4_N)_2_[Mo^IV^O(qpdt)_2_] (**18**) (Figure 4) was thus synthesized and fully characterized including via X-ray crystallography, showing that it shares a number of structural properties with the active site of FDHs. It proved to be quite a good catalyst for both the electro- and photoreduction of protons into hydrogen [39]. However, further analysis showed that **18** was not the active catalyst, but a pre-catalyst. Indeed, under acidic and reductive conditions, the ligand could undergo a ring opening of the pyran cycle [42]. This was unambiguously observed in the case of the [Co^III^Cp(qpdt)] complex **19**, for which the product of electro-reduction **20** could be isolated and characterized. A possible mechanism is illustrated in Scheme 8. This reaction was promoted by the protonation of the quinoxaline cycle. A similar reaction was also observed when the protected ligand **17** was treated with sodium dithionite as a source of electrons. 

**Scheme 9:**
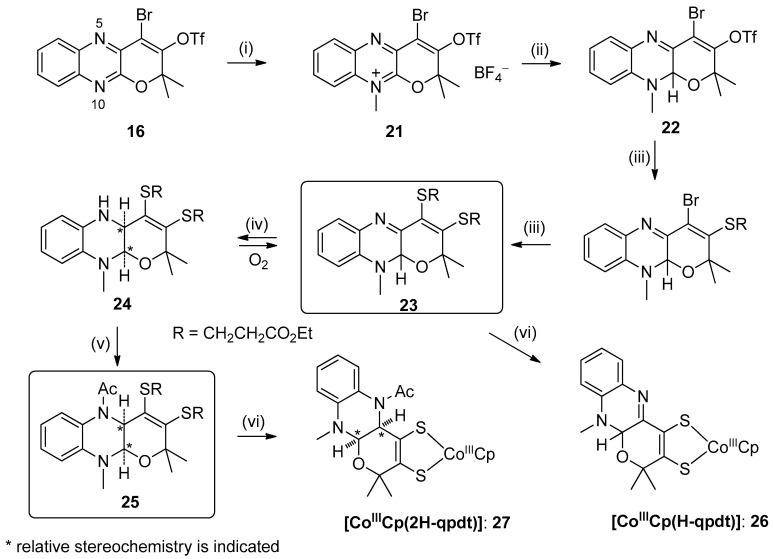
Synthesis of dithiolene ligands **23**, **25**, and complexes [Co^III^Cp(H-qpdt)] **(26)** and [Co^III^Cp(2H-qpdt)] **(27)**. Conditions: (i) (Me_3_O)(BF_4_); (ii) Me_4_NBH(OAc)_3_; (iii) Pd(dba)_2_ (10 %), Xantphos (10 %), HSCH_2_CH_2_CO_2_Et, *i*Pr_2_NEt; (iv) Pd_2_(dba)_3_ (15 %), Xantphos (30 %), HSCH_2_CH_2_CO_2_Et, *i*Pr_2_NEt; (v) NaBH_3_(CN), AcOH; (vi) AcCl, *i*Pr_2_NEt; (vii) CoCpI_2_(CO).

These observations emphasized the necessity to better mimic MPT by reducing the pyrazine cycles. In order to obtain such molecules, we decided to work with the tricyclic skeleton previously described (Scheme 9). Indeed, we showed that obtaining such a motif was the key step in the synthesis. Moreover, these molecules could be easily obtained on a 10 g scale and, as such, constituted a good basis to develop new synthesis. Starting with molecule **16**, the reduction of the first imine on the pyrazine cycle was possible by the activation of N10 with a methylation reaction [43]. The iminium obtained, **21**, could be easily reduced by NaBH(OAc)_3_ to give **22**. Then, following the same methodology as for qpdt, the protected dithiolene moiety was introduced, leading to molecule **23**, with a partially reduced pyrazine cycle. The reduction was completed by treatment with NaBH_3_CN to give the secondary amine **24**, followed by acylation, in order to avoid the reoxidation of the obtained amine, leading to **25**. Interestingly, the two protons of the junction of the pyrazine and pyran cycles in **25** adopt a *cis* configuration (racemic mixture), as is the case, with R, R absolute configuration, in MPT.

**Figure 5:**
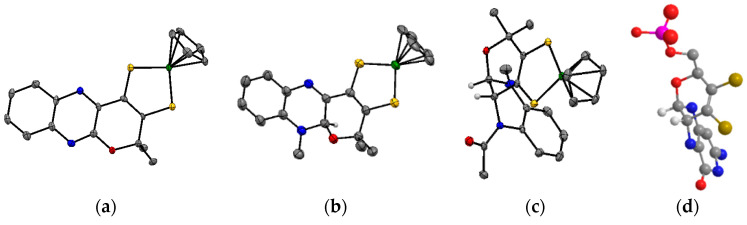
Structure representations of (**a**) [Co^III^Cp(qpdt)] **19**, (**b**) [Co^III^Cp(H-qpdt)] **26**, and (**c**) [Co^III^Cp(2H-qpdt)] **27** and (**d**) Mo^IV^(MPT)_2_ 3D-simplified representation of an MPT ligand in FDHs.

Since the two redox states of the pyrazine cycles of **23** and **25** could be found in nature, they were used for further complexation. Following Joule and Garner’s methodology [26], [Co^III^Cp(dithiolene)] complexes were first prepared in order to structurally characterize the ligands. The structures of the different complexes obtained are shown in Figure 5. As expected, the ligand went from a fully planar structure in qpdt to a bent structure with a significant angle between the pyrazine and the pyran cycle in 2H-qpdt. As a comparison, a 3D representation of ligand MPT in FDHs is shown in Figure 5d and underlines the structural similarity between 2H-qpdt and MPT. The electronic properties were assessed thanks to NMR, UV-visible spectroscopy, and electrochemistry, which showed that with the reduction of the pyrazine cycle, the ligand has a more electron-donating effect on the metal. In particular, 2H-qpdt was much more donating than H-qpdt, which is in good agreement with the hypothesis that the co-existence of both reduced states in natural active sites helps the modulation of their redox activities.

More interestingly, both K_2_[Mo^IV^O(H-qpdt)_2_] (**28**) and (Bu_4_N)_2_[Mo^V^O(2H-qpdt)_2_] (**29**) (Figure 4) could be prepared, constituting the closest mimics of the FDH active site obtained so far. K_2_[Mo^IV^O(H-qpdt)_2_] (**28**) could be structurally characterized, confirming the structure of the ligand found in the structure of [Co^III^Cp(H-qpdt)] **26** (Figure 5b). Two different structures were obtained, one with the dithiolene chelate in a *trans* orientation with respect to the MoS4 core and one with a *cis* orientation. Unfortunately, no crystal structure of (Bu_4_N)_2_[Mo^V^O(2H-qpdt)_2_] (**29**) could be obtained, probably due to the existence of many stereoisomers. Indeed, since 2H-qpdt exists in the form of a mixture of two enantiomers and the two ligands could be *cis*- or *trans*-oriented with respect to the MoS_4_ core, a mixture of seven stereoisomers was expected. Nevertheless, indirect evidence led us to propose that the structure of 2H-qpdt around the metal is similar to the one found in [Co^III^Cp(2H-qpdt)] **27** (Figure 5c). Additionally, (Bu_4_N)_2_[Mo^V^O(2H-qpdt)_2_] (**29**, Figure 4) was well-characterized by other technics and we could confirm its redox state (Mo^V^) thanks to UV-visible and EPR spectroscopy as well as cyclovoltammetry. 

## 4. Functional Models

Until now, most dithiolene complexes studied for their catalytic activity have been tested for the reduction of protons into H_2_ [40,44,45,46,47,48,49,50,51]. In the first part, we will focus on a few examples of tungsten or molybdenum-bis(dithiolene) complexes that were shown to react with carbon dioxide. In the second part, we will discuss metal dithiolene complexes, with other metal ions than molybdenum or tungsten, which catalyze the reduction of CO_2_. Finally, we will also present those active complexes containing tungsten or molybdenum, but without sulfur-based ligands.

### 4.1. Mo/W-(Dithiolene)_2_ Complexes

#### 4.1.1. Equimolar Reaction with CO_2_

**Scheme 10:**
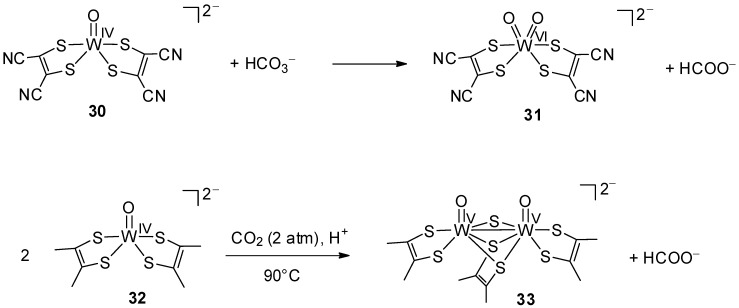
Non-catalytic reduction of CO_2_ by tungsten bis-dithiolene complexes.

The first reaction between a tungsten bis-dithiolene complex and CO_2_ was described by Sarkar and Das [52]. They reported that [W^IV^O(mnt)_2_]^2–^ (**30**) could slowly react with bicarbonate to give formate and [W^VI^O_2_(mnt)_2_]^2–^ (**31**). The yield in formate was 55% based on **30** (Scheme 10). 

Kim and co-workers also described an equimolar reaction between the tungsten complex [W^IV^O(Me_2_C_2_S_2_)_2_]^2–^ (**32**) and CO_2_ which led to formate [53]. This reaction was accompanied by the formation of a triply bridged dinuclear W(V) complex (**33**) (Scheme 10).

#### 4.1.2. Catalytic Reduction of CO_2_

**Scheme 11:**
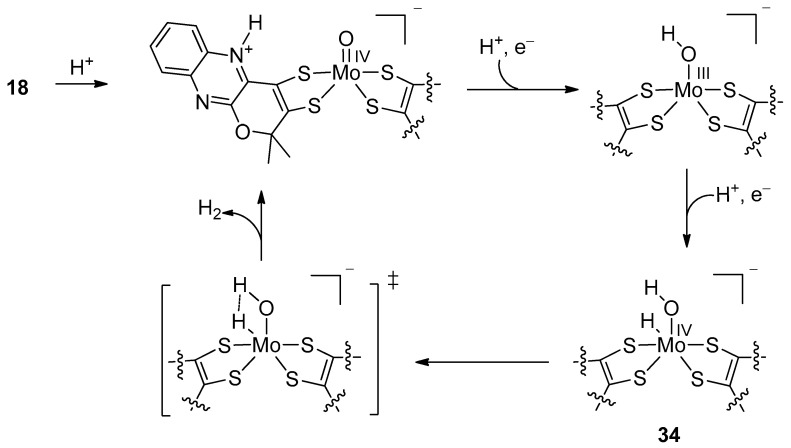
Proposed catalytic cycle for proton reduction by complex **18**.

The three complexes [Mo^IV^O(qpdt)_2_]^2–^ (**18**)_,_ [Mo^IV^O(H-qpdt)_2_]^2–^ (**28**), and [Mo^V^O(2H-qpdt)_2_]^−^ (**29**) (Figure 4) were tested for the photocatalytic reduction of CO_2_ [43]. Catalytic CO_2_ reduction activity was assessed under photochemical conditions, using [Ru(bpy)_3_]_2_^+^ as a photosensitizer (PS), BIH (1,3-dimethyl-2-phenyl-2,3-dihydro-1H-benzoimidazole) as the sacrificial electron donor, and CH_3_CN/TEOA (triethanolamine) in a 5:1 ratio as the solvent, saturated with CO_2_. Complex **18** was highly selective for proton reduction into H_2_, while complexes **28** and **29** showed the ability to catalyze CO_2_ reduction into a mixture of formic acid and carbon monoxide. The most selective complex was complex **29**, with a much larger proportion of CO_2_-derived products accounting for almost 60% (39% formate and 19% CO) and a total TON of 210 after 15 h. Complex **28** was less active (TON= 95) and less selective (53% H_2_). Thus, the redox state of the central pyrazine ring seems to be determinant in selectively catalyzing the reduction of CO_2_ into formate. While FDHs are highly selective for CO_2_ reduction and do not produce any H_2_ during catalysis, the FDH mimics discussed here are also good catalysts for proton reduction. DFT calculations suggested that the axial oxo ligand played a key role in driving this reaction (Scheme 11). Indeed, the protonation of the reduced complex Mo(O) generates a doubly protonated intermediate Mo(OH)(H) **34**, in which one proton bound to the O ligand is very well positioned to react with the Mo-H hydride species, thus generating H_2_, in competition with this hydride reacting with CO_2_ to form formic acid [40]. It is interesting to note that, in contrast, the enzyme has different mainly S-based axial ligands (Figure 2). We made the tentative proposition that this axial coordination is critical for controlling selectivity. However, this requires the functional characterization of appropriate mimics (as shown in Scheme 2).

To date, these complexes, which are the closest mimics of the active site of FDHs, are the only Mo/W-dithiolene complexes able to catalyze the reduction of CO_2_. It is quite interesting to note that not only formate could be obtained, but also that the most selective and active catalyst toward the formation of formate is the one bearing the closest mimic of the natural MPT-ligand. 

**Figure 6:**
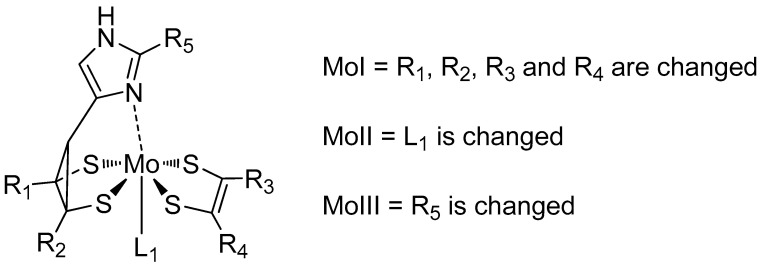
Some Mo-dithiolene complexes potentially interesting for CO_2_ reduction according to the DFT calculations.

Recently, Kumar et al. suggested, thanks to DFT calculations, that adding an imidazole moiety close to the axial position of the metal center could ease the reduction of CO_2_ into formate (Figure 6) [54]. This could be an interesting modification; however, in this purely theoretical work, one of the ligands is not a dithiolene but only a disulfide chelate. 

### 4.2. Mo/W-Cu Complexes, Models of CO-Dehydrogenases

**Figure 7:**
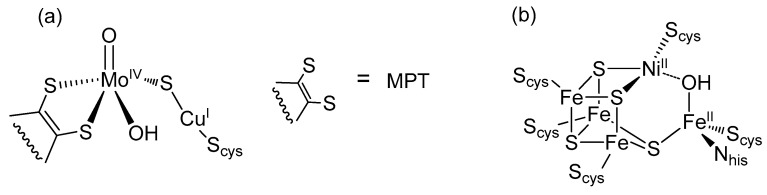
Active sites of (**a**) [MoCu]CODHs with M = Mo and (**b**) [NiFe]CODHs.

Here, we describe bimetallic complexes containing an Mo/W-dithiolene subunit coupled to a mononuclear copper subunit. While the first component mimics the FDH active site, the combination of both metal centers is reminiscent of the active site of CO-dehydrogenase (CODHs). CODH is another metalloenzyme able to reversibly reduce CO_2_ into CO. There are two major families of CODHs: [Mo-Cu] [55] and [Ni-Fe] [56] CODHs. The former contains a heterobimetallic Mo-Cu active site in which the two metal ions are bridged by a sulfide ion, the Mo ion also being coordinated by the MPT ligand and an oxo/hydroxo moiety, and Cu(I) completing its coordination with a cysteinate from the polypeptide chain (Figure 7a); the latter contains a NiFe_4_ cluster (Figure 7b), in which the Ni atom is used as the redox center and the pending Fe atom of the cluster is used exclusively as a Lewis acid to activate CO_2_ and facilitate the cleavage of one of the C-O bonds.

**Figure 8:**
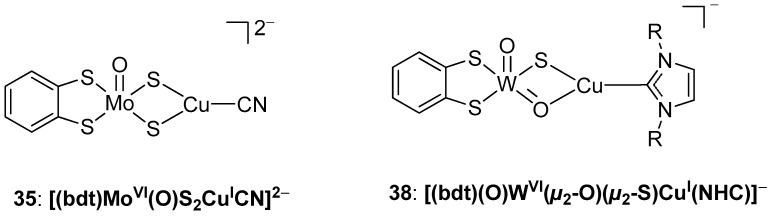
Two model complexes of [MoCu]CODHs.

**Scheme 12:**
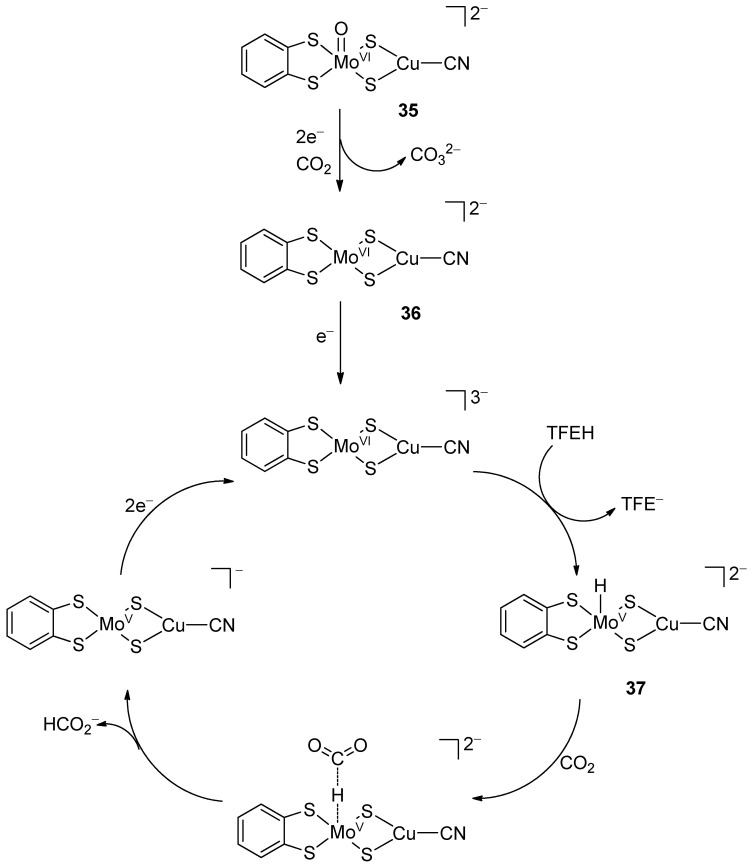
Proposed mechanism of CO_2_ electroreduction catalyzed by [Mo^VI^(O)S_2_(bdt) Cu^I^CN]^2−^ **35**, based on spectroscopic studies and DFT calculations. TFEH= Trifluoroethanol.

Few synthetic dithiolene Mo/W dinuclear complexes have been explored as catalysts for catalytic CO_2_ reduction. The complex [(bdt)Mo^VI^(O)S_2_Cu^I^CN]^2–^ (**35**, Figure 8) has been studied by our group as a catalyst for CO_2_ electroreduction. As a result, complex **35** proved stable during CO_2_ electroreduction in acetonitrile in the presence of a source of protons, and formic acid was obtained as the major product (faradaic efficiency: 70–75%) together with H_2_ (20%) and very small amounts of CO. Spectroscopic studies and DFT calculations (Scheme 12) proved that the complex was, in fact, just a pre-catalyst. Indeed, the first reaction was the loss of the oxo-ligand by a two-electron reduction via an efficient oxo-transfer to CO_2_, leading to carbonate, which eventually dissociates to give **36**, as shown by in situ infra-red spectroelectrochemistry. The release of this coordination site allowed, after another reduction, the formation of a metal-hydride complex **37** which promoted the reduction of CO_2_ into formate [57] (Scheme 12).

This complex is so far the only mimic of [Mo-Cu]CODH to have the ability to catalyze the reduction of carbon dioxide. However, the difference in selectivity between this synthetic model (generating formate) and the enzyme (generating CO) still remains to be explained. 

From that perspective, it is interesting to mention the work of Mankad’s group [58]. They synthesized the [(bdt)(O)W(*µ*_2_-S)(*µ*_2_-O)Cu(NHC)]^−^ complex (**38**, Figure 8) as a synthetic model (NHC = N-heterocyclic carbene). With this complex, they failed to detect any oxidation of carbon monoxide. Supported by DFT calculations, they explained that this absence of reactivity was due to the two *μ*_2_-O and *μ*_2_-S bonds between W and Cu. Indeed, in the natural active sites, the backbone of the enzyme forces the two metals to be far apart from each other, allowing only one *μ*_2_-S bridge between the two atoms, thus creating a frustrated Mo^VI^O/Cu^I^ Lewis pair. These features seemed to be mandatory to obtain CO oxidation. 

### 4.3. Other Mo/W Complexes

Several Mo/W complexes that do not contain any dithiolene ligand have been described as catalysts for the reduction of CO_2_. Even if these complexes are structurally quite different from the active site of FDH, they can bring interesting insights into the role of these metal centers in the reduction of CO_2_. 

**Figure 9:**
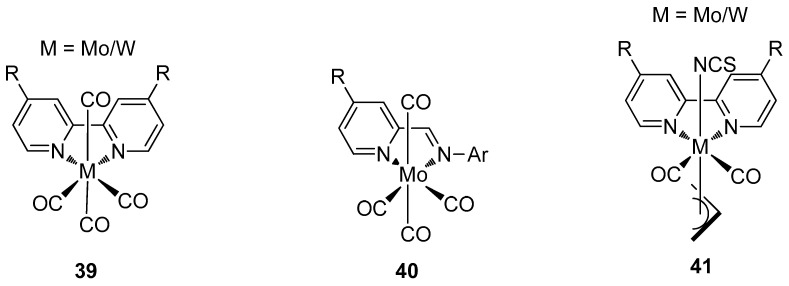
Some Mo/W-based complexes active in CO_2_ reduction.

Based on the previous work on manganese complexes, Kubiak’s group studied the bipyridine-Mo/W(CO)_4_ complex **39** (Figure 9) [59]. After the loss of one carbon monoxide ligand, the active catalyst was formed and could directly reduce CO_2_ into CO. These complexes were very selective toward the formation of CO with a faradaic yield of close to 100%. However, their activity was low with an important overpotential. These results were confirmed by cyclovoltammetry and spectroscopic studies by Tory et al. [60]. Other diamine complexes such as **40** [61] and **41** [62] (Figure 9) have been tested but were too unstable to efficiently catalyze the reduction of CO_2_. Finally, Grice and Saucedo showed that the presence of a non-innocent ligand was not mandatory to promote the reduction of CO_2_, using hexacarbonyl Mo and W complexes [63]. These complexes were functioning at a similar overpotential to produce CO but were less selective with the formation of formate in the presence of water. 

### 4.4. Ni(dithiolene)_2_ Complexes

**Figure 10:**
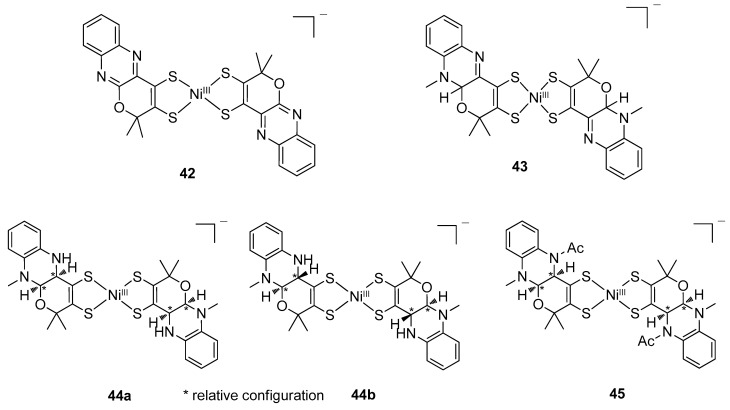
Structures of complexes [Ni^III^(qpdt)_2_]^−^ (**42**), [Ni^III^(H-qpdt)_2_]^−^ (**43**), and [Ni^III^(2H-qpdt)_2_]^−^ (**45**); the chemical reduction of **43** gave **44a** and the electroreduction of **43** gave **44b**.

As mentioned earlier, the Ni center of [Ni-Fe]-CODH, in a tetra-sulfur environment, is the only redox-active metal in the cluster (Figure 7). For this reason, we considered [Ni(dithiolene)_2_] complexes as a reasonable bioinspired complex for mimicking this active site. The availability of the qpdt ligand afforded the possibility to prepare the complex (Bu_4_N)[Ni^III^(qpdt)_2_] (**42**) in which the Ni ion is tetracoordinated in an S_4_ environment with a square planar geometry (Figure 10) [64]. We showed by electrolysis that in the presence of trifluoroethanol as a proton source, this complex was able to promote the reduction of CO_2_ into formate as the major product (60%), together with small amounts of CO and H_2_. However, these results could only be obtained on a mercury electrode. This observation was reminiscent of the case of [Ni(cyclam)]^2+^, and this might originate from favorable transient interactions between the complex and the Hg surface. However, it is very likely that this complex was only a pre-catalyst and that ligand pyran ring opening occurred during electro-reduction. For this reason, we also assessed Ni-bisdithiolene complexes with dithiolene versions with a fully reduced pyrazine ring (Figure 10) [65]. (Bu_4_N)[Ni^III^(H-qpdt)_2_] (**43**) was first prepared, and **44a** and **44b** were obtained via the chemical and electrochemical reduction of **42**, respectively. (Bu_4_N)[Ni^III^(2H-qpdt)_2_] (**45**) was also obtained using the ligand 2H-qpdt. All these complexes catalyzed the reduction of CO_2_ to formate as the major product. Complex **44b** proved to be the most active (larger TONs) and the most selective (FY for formate 70%) among all Ni(bis-dithiolene) complexes studied. This was quite surprising, especially knowing that the only difference between **44a** and **44b** resided in the configuration of the ring junction, *cis* vs. *trans*. It is very likely that this is also the consequence of using an Hg electrode. Thus, we postulate that the noncovalent interactions between the complexes and the Hg surface are strong enough to affect the activity of stereoisomers differently. 

**Scheme 13:**
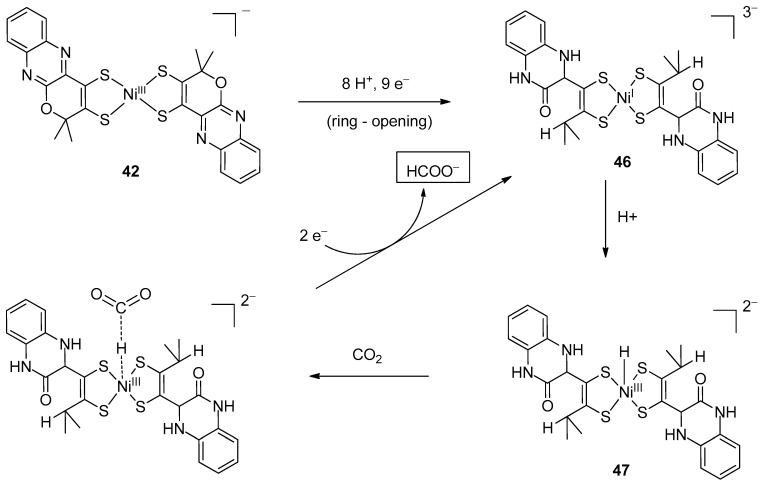
Proposed reaction mechanisms of CO_2_ reduction by complex **42** to formate.

The mechanism of CO_2_ reduction by complex **42** was studied by the DFT calculations [64]. Complex **42** was the pre-catalyst and under electrolysis, the ring-opening reaction first took place to produce **46**, which gave, after protonation, the key intermediate Ni^III^-H **47**. After the departure of formate, **47** was regenerated by a two-electron reduction process (Scheme 13).

## 5. Conclusions

CO_2_ reduction remains a challenging reaction, and a variety of strategies have been followed in order to optimize both solid and molecular catalysts. However, there is still progress to be made in terms of selectivity, efficiency, and stability. The bio-inspired approach, consisting of the design of metal complexes mimicking enzyme active sites, is an original approach that has been exploited only very recently and is still in its infancy. FDHs and CODHs are a family of enzymes that represent remarkable targets for understanding CO_2_ binding, activation, and transformation into formate and CO, respectively. Unfortunately, these enzymes are difficult to study structurally and mechanistically, in particular because of their extreme sensitivity towards O_2_. Bioinspired complexes might thus be ideal to understand CO_2_ reduction mechanisms by natural active sites as well as to discover novel catalysts. The very first studies described here nicely demonstrate that FDHs and CODHs mimics can display interesting catalytic activities. However, more work is needed to better incorporate key components of the active sites, such as S-based axial ligands in FDH mimics, single S bridges in Mo-Cu complexes, as well as Ni-(S)n sites in NiFe clusters. This research might result in fascinating synthetic challenges as well as in novel efficient catalysts.

## Data Availability

This review article did not report any new data out of those already published and available in the corresponding references.
